# Effectiveness of typhoid conjugate vaccine against culture-confirmed *Salmonella enterica* serotype Typhi in an extensively drug-resistant outbreak setting of Hyderabad, Pakistan: a cohort study

**DOI:** 10.1016/S2214-109X(21)00255-2

**Published:** 2021-07-21

**Authors:** Mohammad Tahir Yousafzai, Sultan Karim, Sonia Qureshi, Momin Kazi, Hina Memon, Amber Junejo, Zohra Khawaja, Najeeb Ur Rehman, Muhammad Sajid Ansari, Rafey Ali, Ikram Uddin Ujjan, Heera Mani Lohana, Naveed M Memon, Mudassar Hussain, Roohi Nigar, Naor Bar-Zeev, Farah Naz Qamar

**Affiliations:** a Department of Paediatrics and Child Health, Aga Khan University Hospital, Karachi, Pakistan; b The Kirby Institute, University of New South Wales, Sydney, NSW, Australia; c Liaquat University of Medical and Health Sciences, Jamshoro, Pakistan; d Aga Khan Maternal & Child Care Centre, Hyderabad, Pakistan; e Provincial Disease Surveillance Unit, DG Health Sindh Office, Hyderabad, Pakistan; f International Vaccine Access Center, Department of International Health, Johns Hopkins Bloomberg School of Public Health, Baltimore, MD, USA

## Abstract

**Background:**

*Salmonella enterica* serotype Typhi (*S* Typhi) is a major public health problem in low-income and middle-income countries. We aimed to investigate the effectiveness and impact of the typhoid conjugate vaccine Typbar-TCV against *S* Typhi among children in an outbreak setting of extensively drug-resistant (XDR) *S* Typhi in Pakistan.

**Methods:**

This cohort study was done from Feb 21, 2018, to Dec 31, 2019. A census survey of all households located in the Qasimabad and Latifabad subdistricts of Hyderabad, Pakistan, was done at baseline, and 174 005 households were registered in the census. The Typbar-TCV immunisation campaign was initiated at temporary vaccination centres and 207 000 children aged 6 months to 10 years were vaccinated from Feb 21, 2018, to Dec 31, 2018. Social mobilisers informed parents about the vaccination process. Vaccination records were maintained electronically and linked with the household census surveys. Active surveillance for suspected and blood-culture-confirmed *S* Typhi was established in hospitals, clinics, and laboratories to assess the following outcomes: cases of suspected typhoid fever, culture-confirmed *S* Typhi, and antimicrobial resistance. An age-stratified cohort of 1100 vaccinated children was randomly selected from the vaccination registry, tested for Vi-IgG antibodies (data not reported), and followed up fortnightly (via telephone calls or household visits) until Dec 31, 2019, for ascertainment of outcomes during the study period. 20 847 vaccinated and unvaccinated children were randomly selected from the census registry as a quality control cohort and followed up from Oct 1 to Dec 31, 2019, for ascertainment of outcomes. Vaccine effectiveness against suspected, culture-confirmed, and XDR *S* Typhi was calculated.

**Findings:**

23 407 children from the census registry and surveillance system were included in the vaccine effectiveness analysis. 13 436 (57·4%) children were vaccinated, 12 214 (52·2%) were male, and 10 168 (43·4%) were aged 6–59 months. 5378 (23·0%) of 23 407 children had suspected *S* Typhi, among whom 775 (14·4%) had culture-confirmed *S* Typhi and 361 (68·6%) of 526 had XDR *S* Typhi. Vaccine effectiveness was 55% (95% CI 52–57) against suspected *S* Typhi (regardless of culture confirmation), 95% (93–96) against culture-confirmed *S* Typhi, and 97% (95–98) against XDR *S* Typhi.

**Interpretation:**

Typbar-TCV is effective in protecting children against *S* Typhi infection in an outbreak setting, and was able, with moderate deployment, to curtail a major XDR *S* Typhi outbreak in a densely populated setting. The vaccine shows efficacy against *S* Typhi irrespective of antimicrobial resistance.

**Funding:**

Bill & Melinda Gates Foundation.

## Introduction

Typhoid fever is a serious public health issue in low-income and middle-income countries across Africa and south Asia. More than 90% of the morbidity and mortality related to enteric fever occurs in Asia, predominantly among children aged younger than 15 years. In addition to the high disease burden of typhoid fever, multidrug resistance (resistance or intermediate resistance to ampicillin, chloramphenicol, and co-trimoxazole) and extensive drug resistance (multidrug resistance along with resistance to fluoroquinolone and third-generation cephalosporins)^1^, ^2^, ^3^ to *Salmonella enterica* serotype Typhi (*S* Typhi) are a growing threat.^3^

*S* Typhi is mainly acquired from contaminated water, which is now a ubiquitous challenge in many regions as a result of rapid urbanisation. Another important source is person-to-person transmission through food handlers, especially chronic carriers of *S* Typhi.^4^ Improvements in water and sanitation infrastructure and in food safety require major investments, but at present there is little imminent progress anticipated within these domains in endemic countries. Vaccination against *S* Typhi therefore offers the best approach for disease control.

**Research in context**

**Evidence before this study**

We did an advanced literature search on PubMed and Google Scholar using the key terms “TCV” OR “typhoid conjugate vaccine” OR “Typbar-TCV” OR “typhoid vaccine” AND “effectiveness” OR “impact” OR “efficacy” for studies published up to May 30, 2020. Studies unrelated to the typhoid conjugate vaccine were excluded. A double-blind, placebo-controlled, randomised trial of the typhoid conjugate vaccine Vi-rEPA (Vi bound to non-toxic recombinant *Pseudomonas aeruginosa* exotoxin A) in children aged 2–5 years in Vietnam reported that two doses of the vaccine had a protective efficacy of 91·5% (95% CI 77·1–96·6) against culture-confirmed *Salmonella enterica* serotype Typhi (*S* Typhi) at 27 months; however, this vaccine is not prequalified by WHO. Typbar-TCV is the only typhoid conjugate vaccine prequalified by WHO. A phase 3 randomised controlled trial of Typbar-TCV in Nepal reported a 1-year protective efficacy of 82% with a single dose of the vaccine in children aged 9 months to 16 years. However, more studies are needed to establish the real-world impact and effectiveness of Typbar-TCV—ie, as part of a population-based immunisation campaign in an outbreak setting.

**Added value of this study**

In this cohort study, we found that a single dose of Typbar-TCV was highly effective (vaccine effectiveness >90%) against culture-confirmed drug-resistant and culture-sensitive *S* Typhi in children aged 6 months to 10 years in a population-wide campaign setting in Hyderabad, Pakistan. Mass immunisation with Typbar-TCV resulted in a significant reduction in culture-confirmed *S* Typhi cases during the peak monsoon seasons of 2018 and 2019, demonstrating the impact of vaccination in protecting against increased community transmission of *S* Typhi during the peak monsoon season.

**Implications of all the available evidence**

Typbar-TCV is effective against culture-confirmed *S* Typhi in children aged 6 months to 10 years in an outbreak setting in Pakistan and, with only moderate deployment, was able to curtail an extensively drug-resistant *S* Typhi outbreak in a densely populated setting. The vaccine is effective against *S* Typhi irrespective of antimicrobial resistance. Typbar-TCV can therefore be an important tool to control drug-resistant *S* Typhi outbreaks. Furthermore, on the basis of these findings, incorporation of Typbar-TCV into routine immunisation programmes in endemic countries is highly recommended.

Older typhoid vaccines, including a single dose of the intramuscular Vi-polysaccharide and three-dose oral Ty21a vaccine, have suboptimal efficacy, a short duration of protection, and cannot be administered to children younger than 2 years.^5^, ^6^ However, the new typhoid conjugate vaccine (Typbar-TCV; Bharat Biotech International; Hyderabad, India) has shown promising efficacy in phase 2b and phase 3 trials.^7^, ^8^ Moreover, it can be given to children from 6 months of age and is prequalified by WHO.^9^ A human challenge study of Typbar-TCV among adult volunteers in the UK reported a vaccine efficacy of 54·6% against *S* Typhi bacteraemia.^7^ The results of the phase 3 trial of Typbar-TCV in Nepal showed a vaccine efficacy of 81·6% in children aged 9 months to 16 years.^8^ However, population-based observational studies are urgently needed to estimate the effectiveness of Typbar-TCV in settings with antimicrobial resistance.

In November, 2016, the Aga Khan University Hospital, Karachi, Pakistan, identified an emergent extensively drug-resistant (XDR) *S* Typhi outbreak in Hyderabad, Pakistan.^10^ A large-scale vaccination campaign was initiated to interrupt transmission. We aimed to determine the public health impact and effectiveness of Typbar-TCV in preventing blood-culture-confirmed *S* Typhi in children aged 6 months to 10 years, living in the outbreak areas of Hyderabad, Pakistan.

## Methods

### Study design and setting

**Figure 1:**
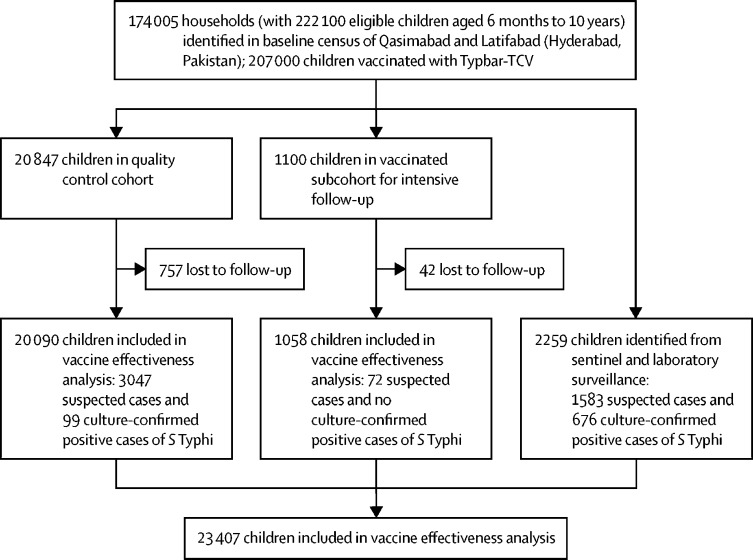
Study flow diagram *S* Typhi=*Salmonella enterica* serotype Typhi. Participants were followed up from the date of the census survey or vaccination until Dec 31, 2019.

This cohort study was done from Feb 21, 2018, to Dec 31, 2019, in the Qasimabad and Latifabad subdistricts of Hyderabad, Pakistan. The total population of Hyderabad is around 2·2 million, with 44% of the population (977 000) living in these two subdistricts.^10^ Hyderabad is composed of a mixture of a middle-class and high-density urban slum population. Details of the study setting have been published,^11^ and details of the study are summarised in figure 1.

### Baseline census and surveillance

We did a census survey of all households located in the Qasimabad and Latifabad subdistricts of Hyderabad. Each household was assigned a unique identifier. On the day of the visit, global positioning system (GPS) coordinates and the number, names, sex, and date of birth of children aged 6 months to 10 years living in each household and the name of the head of the household were recorded. Each child was assigned a unique identifier. Households located in apartments or residential compounds were assigned a single GPS coordinate. Non-responsive households were revisited at the end of the day or a catch-up visit was made on the following day. Following these attempts, non-responding households were assigned an identifier and GPS coordinates, and coded as locked or non-responding. 174 005 households were registered in the census. This census registry served as the data frame linking all subsequent records from the other components of the study, including Typbar-TCV vaccination status during the immunisation campaign, and the surveillance system.

A detailed description of the surveillance system and case definition for suspected and culture-confirmed *S* Typhi has been published.^12^, ^13^ Briefly, we established active surveillance for identification of suspected and blood-culture-confirmed *S* Typhi at 13 sentinel sites, in all major hospitals and clinics of general practitioners (GPs) in the two subdistricts. Patients presenting to these sentinel sites with persistent fever for 3 or more days without any focus of infection were considered to have suspected typhoid fever, whereas those with a positive blood culture for *S* Typhi were recorded as having culture-confirmed *S* Typhi infection. Training on case definitions, the target population, and details of the study was provided to at least one focal physician at each sentinel site. A trained research assistant visited each facility daily to ensure cases were not missed. Patients with suspected *S* Typhi were offered free blood culture testing at the nearest Aga Khan University Hospital clinical laboratory collection point in Hyderabad, Pakistan. We also included cases of culture-confirmed *S* Typhi from two major laboratories in Hyderabad: the clinical laboratory of Aga Khan University Hospital and that of Liaquat University of Medical Health Sciences, Hyderabad, Pakistan. The surveillance system captures the whole population of Latifabad and Qasimabad.

### Participants and procedures

We began a population-based immunisation campaign using Typbar-TCV in children aged 6 months to 10 years living in Qasimabad and Latifabad. All eligible children were offered free, voluntary vaccination, and 207 000 children were vaccinated at temporary vaccination centres from Feb 21, 2018, to Dec 31, 2018.^11^ With every vaccine dose administered, we recorded the recipient's name, age or date of birth (or both), sex, household and child identifier, date of vaccination, injection site, vial batch number, and history of previous typhoid vaccination. The immunisation record was linked to the baseline census registry through unique household and child identifiers.

We randomly selected an age-stratified subcohort of 1100 vaccinated children and followed them up from the day of vaccination until Dec 31, 2019. We used systematic random sampling with replacement to select every fifth child in each age group (150 children each aged 6–11 months, 12–23 months, 24–35 months, 36–47 months, and 48–59 months, and 70 children each aged 60–71 months, 72–83 months, 84–95 months, 96–107 months, and 108–120 months) visiting the temporary vaccination centres. This cohort was periodically followed up (at 28–42 days, 6 months, 1 year, and 2 years) for serial measurement of serum antibody concentrations against *S* Typhi Vi-IgG (data not reported), and fortnightly telephone calls were made by the research assistants to enquire about history of illness, including fever or admission to hospital, or both, during the past 2 weeks. Data on the immunogenicity of Typbar-TCV will be published at a later date, once all blood samples have been analysed for Vi-IgG concentrations. Where febrile illness was identified, the research medical officer visited the household to examine the child and collect further clinical details, a hospital discharge summary, and laboratory tests (if available), and to establish whether the child fulfilled the case definition for suspected typhoid fever or culture-confirmed *S* Typhi. Any child with a fever for 3 or more days at the time of the telephone call was provided free blood culture testing at the nearest Aga Khan University Hospital clinical laboratory collection point. Cases were cross-checked with surveillance data to avoid duplicates.

We randomly selected another quality control cohort of 20 847 children using simple random sampling without replacement from the baseline census and followed them up until Dec 31, 2019. From Oct 1 to Dec 31, 2019, trained research assistants visited the households to interview parents and collect information about the history of fever, suspected typhoid fever, and laboratory testing for *S* Typhi (blood culture, Typhidot), and hospital admissions from the date of baseline census or Typbar-TCV vaccination until the date of the interview. Information about age, sex, and vaccination status was available from the census registry. Information about laboratory testing, prescriptions or hospital discharge summary (if available), or both, was also recorded. Cases were cross-checked with surveillance system records to remove any duplicates. Cases were defined as suspected typhoid fever or culture-confirmed *S* Typhi on the basis of clinical diagnosis by clinicians working at the sentinel hospitals or, where applicable, laboratory confirmation of blood cultures. The quality control cohort thus provided evidence that cases of suspected typhoid fever and culture-confirmed *S* Typhi were not missed as outcomes by the surveillance system.

We developed an electronic data capture programme using Java structured query language (SQL) platforms as a database for real-time data collection. Data were synced to a central server at the Aga Khan University. Dedicated staff (NUR and RA) carried out quality assurance and quality control checks to identify illogical responses and a weekly quality assurance and quality control report was provided to the research supervisor (SK) in Hyderabad for field verification. Children who were missed during the baseline census were assigned new registration numbers whenever they were encountered by field staff, and their records were assigned to their respective household identifier. We linked datasets from the typhoid surveillance, vaccinated cohort, quality control cohort, and vaccination registry with the baseline census using the household identifier and children's unique identifiers.

Ethical approval was obtained from the ethical review committee of Aga Khan University Karachi (ERC number 2020-1563-14158) and the National Bioethics Committee of Pakistan (4-87/NBC-323/18/551). Written informed consent was obtained from one of the parents or legal guardians at the time of enrolment into the study.

### Outcomes

The outcomes of interest were the development of suspected typhoid fever (irrespective of blood culture), culture-confirmed *S* Typhi (irrespective of antimicrobial resistance), culture-confirmed XDR *S* Typhi, and non-XDR *S* Typhi (sensitive to ceftriaxone with or without multidrug resistance or fluoroquinolone resistance, or both) among age-eligible unvaccinated children and those vaccinated with a single dose of Typbar-TCV.

### Statistical analysis

We analysed data using Stata, version 15. The vaccinated cohort and quality control cohort were merged with the surveillance records for this analysis. Since the vaccination campaign and baseline registration of households occurred on the same day in each neighbourhood, the person-time at risk for each child was calculated from the date of vaccination (for vaccinated children) or the date of baseline registration (for unvaccinated children) to the date of suspected typhoid fever or culture-confirmed *S* Typhi (for children with an outcome) or the end of study follow-up (Dec 31, 2019) or death or loss to follow-up, whichever occurred first. Incidence rates with their corresponding incidence rate ratio (IRR) and 95% CIs were calculated. We excluded any cases of typhoid fever that occurred less than 28 days after vaccination from the calculation of vaccine effectiveness. Vaccine effectiveness, with 95% CIs, against culture-confirmed, culture-negative suspected, and XDR *S* Typhi was calculated as follows: 100 × (1–IRR). Cox regression analysis was done to measure the hazard ratio (HR) of developing culture-confirmed *S* Typhi when adjusted for age, sex, and residential area. Kaplan-Meier curves were also plotted to compare the risk of developing culture-confirmed *S* Typhi among vaccinated versus unvaccinated children, children aged 6–59 months versus those aged 60–120 months, children living in Latifabad versus those living in Qasimabad, and being male versus female. Two-sided p values were calculated with the log-rank test to estimate statistically significant differences between the Kaplan-Meier curves for each variable. We measured the impact of Typbar-TCV by calculating the differences in quarterly cumulative incidence of culture-confirmed *S* Typhi before (2017) and following (2018–19) the vaccination campaign. No imputation was done for missing information, and participants with missing details for any variable were labelled as “unknown”.

The sample size was calculated with OpenEpi, version 3, for cohort design. Assuming the incidence of culture-confirmed *S* Typhi among unvaccinated children aged 6 months to 15 years is 455 per 100 000,^3^ and assuming 50% vaccine coverage, in order to detect a vaccine effectiveness of at least 55% against culture-confirmed *S* Typhi^8^ with 80% power and 95% CIs, using the χ^2^ test with continuity correction and after adjusting for a non-response rate of 15%, the required minimum sample size was 20 847 for the quality control cohort. We did not do any sample size calculation for the subset of the vaccinated cohort for continuous fortnightly follow-ups. The vaccinated subcohort was arbitrarily selected primarily for immunogenicity assessment at several timepoints and periodically followed up for ascertainment of an outcome.

### Role of the funding source

The funder of the study had no role in study design, data collection, data analysis, data interpretation, or writing of the report.

## Results

24 206 children from the census registry and surveillance system were selected for this study; after removing 799 (3·3%) non-responders, 23 407 children were included in the vaccine effectiveness analysis (figure 1). Of these 23 407 children, 13 436 (57·4%) were vaccinated with Typbar-TCV (table 1). The Latifabad subdistrict accounted for 13 695 (58·5%) of children included in the study and the remainder were from Qasimabad. 12 214 (52·2%) children were male, and 10 168 (43·4%) were aged 6–59 months. 5378 (23·0%) of 23 407 children were identified to have suspected typhoid fever, of whom 2496 (46·4%) were tested for blood culture and 775 (31·0%) of 2496 were positive for *S* Typhi. Information about antimicrobial sensitivity was available for 655 (84·5%) of these 775 children, of whom 444 (57·3%) had XDR *S* Typhi.

**Table 1:** Descriptive characteristics of participants included in vaccine effectiveness analysis (n=23 407)

		**Vaccinated (n=13 436)**	**Unvaccinated (n=9971)**	**Total (n=23 407)**
Study component				
	Surveillance registry	216 (1·6%)	2043 (20·5%)	2259 (9·7%)
	Case cohort	12 162 (90·5%)	7928 (79·5%)	20 090 (85·8%)
	Vaccinated subcohort	1058 (7·9%)	..	1058 (4·5%)
Area of residence in Hyderabad, Pakistan				
	Qasimabad	5382 (40·1%)	4330 (43·4%)	9712 (41·5%)
	Latifabad	8054 (59·9%)	5641 (56·6%)	13 695 (58·5%)
Sex				
	Male	7010 (52·2%)	5204 (52·2%)	12 214 (52·2%)
	Female	6426 (47·8%)	4767 (47·8%)	11 193 (47·8%)
Age groups				
	6–59 months	5521 (41·1%)	4647 (46·6%)	10 168 (43·4%)
	60–120 months	7915 (58·9%)	5314 (53·4%)	13 239 (56·6%)
Overall typhoid fever cases		1969 (14·7%)	3409 (34·2%)	5378 (23·0%)
Blood culture tests (n=5378)				
	Yes	320/1969 (16·3%)	2176/3409 (63·8%)	2496/5378 (46·4%)
	No	1649/1969 (83·7%)	1233/3409 (36·2%)	2882/5378 (53·6%)
Blood culture result (n=2496)				
	Negative	273/320 (85·3%)	1448/2176 (66·5%)	1721/2496 (69·0%)
	Positive for *S* Typhi	47/320 (14·7%)	728/2176 (33·5%)	775/2496 (31·0%)
XDR status (n=775)				
	Non-XDR	6/47 (12·8%)	205/728 (28·2%)	211/775 (27·2%)
	XDR	18/47 (38·3%)	426/728 (58·5%)	444/775 (57·3%)
	Results not known	23/47 (48·9%)	97/728 (13·3%)	120/775 (15·5%)

Data are n (%) or n/N (%). *S* Typhi*=Salmonella enterica* serotype Typhi. XDR=extensively drug-resistant.

Overall, the incidence of suspected typhoid fever was 9490 (95% CI 9361–9619) per 100 000 person-years among vaccinated children and 20 886 (20 566–21 207) per 100 000 person-years among unvaccinated children. The IRRs comparing vaccinated and unvaccinated children with suspected (regardless of culture) *S* Typhi, culture-negative suspected *S* Typhi, culture-confirmed *S* Typhi, XDR *S* Typhi, and non-XDR *S* Typhi are shown in table 2. Vaccine effectiveness was 55% (95% CI 52–57) against suspected *S* Typhi (regardless of culture), 44% (40–47) against culture-negative suspected *S* Typhi, 95% (93–96) against culture-confirmed *S* Typhi, 97% (95–98) against XDR *S* Typhi, and 98% (95–99) against non-XDR *S* Typhi.

**Table 2:** Distribution of vaccination status and vaccine effectiveness against *S* Typhi

		**Number of participants (n)**	**At-risk population (n)**	Total person-time at risk, years*	***S* Typhi incidence; number of cases per 100 000 population (95% CI)**	***S* Typhi incidence; number of cases per 100 000 person-years (95% CI)**	***S* Typhi incidence rate ratio (95% CI)**	Vaccine effectiveness*(95% CI)
**Both culture-confirmed and suspected *S* Typhi cases**								
Age 6–59 months								
	Vaccinated	940	5521	8646	17 025·9 (16 034·4–18 017·4)	10 872·2 (10 643·0–11 101·3)	0·50 (0·46–0·54)	50·0 (45·8–53·9)
	Unvaccinated	1653	4647	7599	35 571·3 (34 194·9–36 947·8)	21 752·1 (21 263·0–22 241·1)	..	..
Age ≥5 years								
	Vaccinated	1029	7915	12103	13 000·6 (12 259·7–13 741·5)	8502·2 (8350·7–8653·6)	0·42 (0·39–0·46)	57·8 (54·4–60·9)
	Unvaccinated	1756	5324	8722	32 982·7 (31 719·8–34 245·6)	20 132·0 (19 709·5–20 554·5)	..	..
Overall								
	Vaccinated	1969	13436	20749	14 654·7 (14 056·7–15 252·7)	9489·7 (9360·6–9618·9)	0·45 (0·43–0·48)	54·6 (52·0–57·0)
	Unvaccinated	3409	9971	16322	34 189·1 (33 258·1–35 120·2)	20 886·3 (20 565·9–21 206·7)	..	..
**Suspected typhoid fever cases**								
Age 6–59 months								
	Vaccinated	918	5521	8646	16 627·4 (15 645·3–17 609·6)	10 617·7 (10 393·9–10 841·5)	0·62 (0·57–0·67)	38·1 (32·6–43·2)
	Unvaccinated	1304	4647	7599	28 061·1 (26 769·3–29 352·9)	171 59·5 (16 773·7–17 545·3)	..	..
Age ≥5 years								
	Vaccinated	1004	7915	12 103	12 684·8 (11951·6–13 418·0)	8295·6 (8147·8–8443·4)	0·53 (0·48–0·57)	47·5 (43·0–51·6)
	Unvaccinated	1377	5324	8722	25 864·0 (24 687·8–27 040·3)	15 786·9 (15 455·6–16 118·2)	..	..
Overall								
	Vaccinated	1922	13 436	20 749	14 304·9 (13 712·8–14 896·9)	9263·2 (9137·2–9389·2)	0·56 (0·53–0·60)	43·6 (40·2–46·8)
	Unvaccinated	2681	9971	16 322	26 888·0 (26 017·7–27 758·3)	16 426·0 (16 174·0–16 678·0)	..	..
**Culture-confirmed *S* Typhi cases**								
Age 6–59 months								
	Vaccinated	22	5521	8646	398·5 (232·3–564·7)	254·5 (249·1–259·8)	0·06 (0·03–0·09)	94·5 (91·5–96·6)
	Unvaccinated	349	4647	7599	7510·2 (6752·4–8268·0)	4592·5 (4489·3–4695·8)	..	..
Age ≥5 years								
	Vaccinated	25	7915	12 103	315·9 (192·2–439·5)	206·6 (202·9–210·2)	0·05 (0·03–0·07)	95·2 (92·9–97·0)
	Unvaccinated	379	5324	8722	7118·7 (6428·0–7809·4)	4345·1 (4253·9–4436·3)	..	..
Overall								
	Vaccinated	47	13436	20 749	349·8 (250·0–449·6)	226·5 (223·4–229·6)	0·05 (0·04–0·07)	94·9 (93·2–96·3)
	Unvaccinated	728	9971	16 322	7301·2 (6790·5–7811·8)	4460·3 (4391·9–4528·8)	..	..
**XDR *S* Typhi cases**								
Age 6–59 months								
	Vaccinated	14	5521	8646	253·6 (120·9–386·2)	161·9 (158·5–165·3)	0·06 (0·03–0·10)	94·4 (90·4–97·0)
	Unvaccinated	220	4647	7599	4734·2 (4123·6–5344·8)	2895·0 (2829·9–2960·1)	..	..
Age ≥5 years								
	Vaccinated	4	7915	12 103	50·5 (1·0–100·1)	33·1 (32·5–33·6)	0·01 (0·00–0·04)	98·6 (96·4–99·6)
	Unvaccinated	206	5324	8722	3869·3 (3351·2–4387·3)	2361·7 (2312·2–2411·3)	..	..
Overall								
	Vaccinated	18	13 436	20 749	134·0 (72·1–195·8)	86·8 (85·6–87·9)	0·03 (0·02–0·05)	96·7 (94·7–98·0)
	Unvaccinated	426	9971	16 322	4272·4 (3875·4–4669·3)	2610·0 (2570·0–2650·1)	..	..
**Non-XDR *S* Typhi cases**								
Age 6–59 months								
	Vaccinated	2	5521	8646	36·2 (−14·0–86·4)	23·1 (22·6–23·6)	0·02 (0·00–0·07)	98·1 (93·1–99·8)
	Unvaccinated	94	4647	7599	2022·8 (1618·0–2427·6)	1237·0 (1209·1–1264·8)	..	..
Age ≥5 years								
	Vaccinated	4	7915	12103	50·5 (1·0–100·1)	33·1 (32·5–33·6)	0·03 (0·01–0·07)	97·4 (93·2–99·3)
	Unvaccinated	111	5324	8722	2084·9 (1701·1–2468·7)	1272·6 (1245·9–1299·3)	..	..
Overall								
	Vaccinated	6	13 436	20 749	44·7 (8·9–80·4)	28·9 (28·5–29·3)	0·02 (0·01–0·05)	97·7 (94·9–99·2)
	Unvaccinated	205	9971	16 322	2056·0 (1777·4–2334·5)	1256·0 (1236·7–1275·3)	..	..

IRR=incidence rate ratio. *S* Typhi*=Salmonella enterica* serotype Typhi. XDR=extensively drug resistant.

* Calculated as follows: (1 – IRR) × 100.

**Figure 2:**
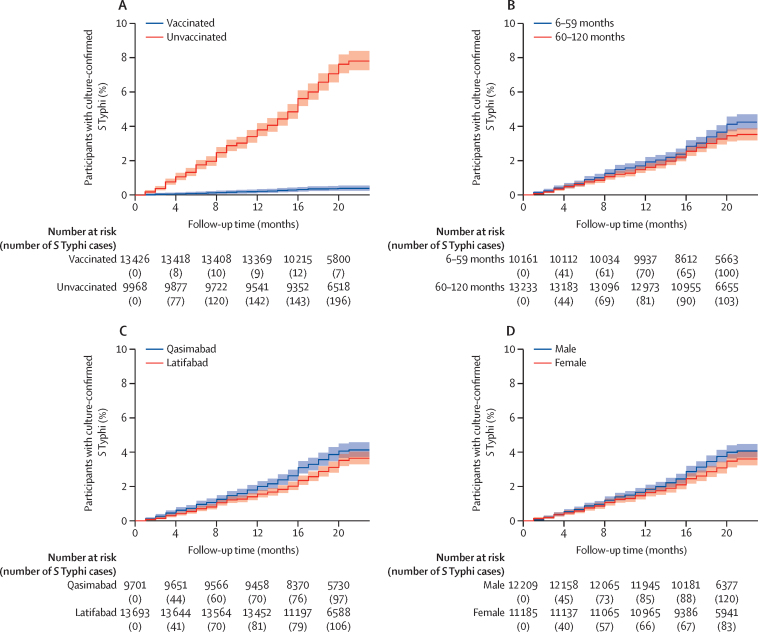
Survival analysis of culture-confirmed *S* Typhi cases (A) Vaccinated versus unvaccinated children. (B) Children aged 6–59 months versus those aged 60–120 months. (C) Area of residence in Hyderabad, Pakistan (Qasimabad *vs* Latifabad). (D) Male versus female cases. *S* Typhi=*Salmonella enterica* serotype Typhi.

The HR of culture-confirmed *S* Typhi among vaccinated children versus unvaccinated children, adjusted for age, sex, and area of residence, was 0·05 (95% CI 0·04–0·07; p<0·0001; table 3). Kaplan-Meier plots also showed a significantly lower risk of culture-confirmed *S* Typhi among vaccinated children than among unvaccinated children (p<0·0001), in children aged 60–120 months than in those aged 6–59 months (p=0·028), in children living in Latifabad versus those living in Qasimabad (p=0·016), and in girls versus boys (p=0·044; figure 2).

**Table 3:** Univariable and multivariable Cox regression analysis for assessment of culture-confirmed *S* Typhi

	**Univariable hazard ratio (95% CI)**	**p value**	**Multivariable hazard ratio (95% CI)**	**p value**
**Vaccine status**				
Vaccinated	0·05 (0·04–0·07)	<0·0001	0·05 (0·04–0·07)	<0·0001
Unvaccinated	1 (ref)	..	1 (ref)	..
**Age groups**				
6–59 months	1·17 (1·02–1·35)	0·028	1·07 (0·93–1·23)	0·346
60–120 months	1 (ref)	..	1 (ref)	..
**Sex**				
Male	1·16 (1·004–1·33)	0·044	1·15 (1–1·33)	0·050
Female	1 (ref)	..	1 (ref)	..
**Area of residence in Hyderabad, Pakistan**				
Qasimabad	0·84 (0·73–0·97)	0·014	0·88 (0·77–1·02)	0·083
Latifabad	1 (ref)	..	1 (ref)	..

*S* Typhi*=Salmonella enterica* serotype Typhi.

Throughout the epidemic (2017–19), the highest number of non-XDR *S* Typhi cases was reported in 2017, followed by a decline in 2018 and then another rise during July–August, 2019 (appendix p 2). The interrupted time-series plot of XDR and non-XDR *S* Typhi cases showed a slight decline in 2019 compared to 2017 (before the intervention; appendix p 3). Overall, we observed a significant decline in cumulative incidence between 2017 (the reference period) and 2018, and between 2017 and 2019, except during October–December in 2017–19, when a significant rise in incidence was observed (appendix p 4).

## Discussion

As Typbar-TCV was prequalified by WHO in October, 2017,^9^ its post-licensure population impact and effectiveness data have not yet been extensively reported.

This population-based, large-scale, prospective cohort study, done in a setting with an ongoing XDR *S* Typhi outbreak, demonstrates the effectiveness of Typbar-TCV against culture-confirmed *S* Typhi. The effectiveness of Typbar-TCV was 95% against culture-confirmed *S* Typhi and 55% against suspected *S* Typhi (regardless of culture). Vaccine effectiveness against XDR *S* Typhi was 97%. The risk of contracting culture-confirmed *S* Typhi was 95% lower among vaccinated children than among unvaccinated children. The quarterly incidence of *S* Typhi after the vaccination campaign (2018–19) was significantly lower than before the vaccination campaign (2017); however, during the last quarters of 2018 and 2019, incidence of *S* Typhi was higher than in 2017. The most plausible explanation for the rise in cases during this period could be the seasonality of *S* Typhi, as higher numbers of cases—including outbreaks—occur during the monsoon period (May to September), followed by sporadic cases during the rest of the year. Although mass immunisation with Typbar-TCV could substantially reduce the number of *S* Typhi cases during the peak monsoon season, we did not observe a noticeable decline outside of the peak monsoon season in 2018–19. 3-year (2017–19) *S* Typhi surveillance data with the same case definition from the adjacent city of Karachi, Pakistan, where mass immunisation with Typbar-TCV was not initiated until the end of 2019, showed a significantly higher number of *S* Typhi cases in all quarters of 2018–19 compared to 2017,^3^ further demonstrating that the decline in the number of cases during the monsoon periods of 2018–19 in Hyderabad, Pakistan, was not an artifact of fewer rains or mild monsoons during 2018–19 compared to 2017.

Limited data from the use of Typbar-TCV to control a typhoid fever outbreak in Zimbabwe reported a sharp decline in the number of culture-confirmed and suspected *S* Typhi cases among children aged 6 months to 15 years after 3 months of the vaccination campaign. The authors reported culture-confirmed *S* Typhi in 21% of cases before the vaccination campaign compared to none after vaccination with Typbar-TCV.^14^ The study from Zimbabwe did not report vaccine effectiveness or a difference in incidence of *S* Typhi before and after the vaccination campaign and hence cannot be compared with our estimates. Furthermore, the number of culture-confirmed *S* Typhi cases reported from Zimbabwe was small (23 of 109 blood cultures before the immunisation campaign and 0 of 24 blood cultures afterwards), whereas in the present study we established sentinel and laboratory surveillance, and thousands of blood cultures were done to identify culture-confirmed *S* Typhi. In the phase 3 randomised controlled trial in Nepal, 20 019 children aged 6 months to 16 years were enrolled and randomly assigned to receive either a single dose of Typbar-TCV or the meningococcal A vaccine; preliminary data reported a 1-year protective efficacy of TCV of 81·6% (95% CI 58·8–91·8) against culture-confirmed *S* Typhi.^8^ When restricting the analysis to *S* Typhi cases confirmed by blood culture among participants who presented with fever for at least 3 days before blood culture testing, the 1-year protective efficacy further increased to 85·1% (95% CI 49·7–95·6). These results are consistent with our findings from Hyderabad, Pakistan, where the case definition included 3 days of fever before blood culture testing and positivity for *S* Typhi. Likewise, data from a double-blind, randomised, placebo-controlled trial of children aged 2–5 years in Vietnam reported that two doses of another typhoid conjugate vaccine (Vi bound to non-toxic recombinant *Pseudomonas aeruginosa* exotoxin A) had a protective efficacy of 91·5% (95% CI 77·1–96·6) against culture-confirmed *S* Typhi for 27 months.^15^ By contrast, a single-centre, phase 2b trial based on an established human challenge model in the UK enrolled 112 healthy volunteers aged 18–60 years with no history of typhoid fever, typhoid vaccination, or prolonged exposure in a typhoid-fever-endemic region, and participants were either administered Typbar-TCV, the Vi-polysaccharide vaccine, or the meningococcal conjugate vaccine (control group); 1 month after vaccine administration, all participants were exposed to *S* Typhi via oral ingestion and closely assessed for the development of typhoid fever (persistent fever ≥38°C for at least 12 h or *S* Typhi bacteraemia). Based on this study, Typbar-TCV was reported to have an effectiveness of only 55% (95% CI 27–72).^7^ The potential explanation for the relatively lower efficacy of Typbar-TCV in the human challenge study done in the UK could be the enrolment of adult participants from a non-endemic setting without previous exposure to *S* Typhi and applying a more sensitive case definition (including *S* Typhi bacteraemia with or without clinical manifestations), which might not be detectable in an endemic setting. Another explanation for the low efficacy could be the high challenge dose administered after the neutralisation of gastric acid.

Although the findings of the phase 3 trial of Typbar-TCV in Nepal have been reported,^8^ our study demonstrates the real-world effectiveness of the vaccine in the context of an outbreak response in an urban, low-income to middle-income, typhoid-endemic setting. And although the internal validity of randomised controlled trials is important, the generalisability of the findings beyond the study population is always debatable.^16^ Furthermore, the confidence interval of the reported vaccine efficacy in the randomised controlled trial done in Nepal was quite wide, suggesting either low power of the study or a fewer number of cases than expected. By contrast, our cohort study in Hyderabad, Pakistan, was sufficiently powered and thus our findings have greater generalisability and policy implications. This study is also, to the best of our knowledge, the first to report the impact of Typbar-TCV against XDR *S* Typhi.

Key strengths of our study include the use of a cohort study design, establishment of a baseline population census registry, a tight surveillance system, uniform case definition for confirmed and suspected cases, and a large sample size, all of which increase not only the internal but also external validity of our findings. Moreover, to our knowledge this is the first study to report the effectiveness of Typbar-TCV in children as young as 6 months.

This study provides important evidence that has implications for vaccination campaigns to control typhoid fever outbreaks. Furthermore, although Pakistan has sub-optimal vaccination coverage for routine immunisation,^17^ the fact that this study showed a vaccine effectiveness of more than 90% with a modest coverage of 57% is reassuring for Pakistan and other countries facing similar challenges relating to routine immunisation. Furthermore, this study was done in an outbreak setting where the predominant strain type was XDR, indicating that Typbar-TCV can help counter antimicrobial resistance and is presumably expected to have a concomitant impact on mortality from typhoid fever as well.

This study had several limitations. First, clinicians only started using blood culture as a diagnostic tool for typhoid fever instead of serology in the face of the XDR *S* Typhi outbreak, leading to ascertainment of a higher number of culture-confirmed *S* Typhi cases, and hence the change from serology-based to blood-culture-based diagnoses happened gradually, especially at the start of the vaccination campaign, so our impact assessment (comparing the incidence of *S* Typhi before and after vaccination) might have been biased towards the null, resulting in underestimation of the impact of Typbar-TCV. Second, attendance for blood culture was much lower among vaccinated than among unvaccinated children. This is likely to be due to a vaccine-induced reduction in disease burden, but it is plausible that vaccination itself might confer improved confidence in wellbeing and reduced treatment-seeking behaviour. To mitigate this possibility, we enrolled children independently through the surveillance system without ascertainment of their vaccination status at the time of enrolment, provided a free-of-cost blood testing facility for everyone who fulfilled the suspected case definition, and involved the data management team to electronically link vaccination records. Last, XDR status was unknown for 48·9% of the vaccinated culture-confirmed *S* Typhi cases, compared to 13·3% of unvaccinated cases. This differential misclassification of unknown XDR status (although unknown data were excluded during the analysis) has the potential to bias our vaccine effectiveness estimates against XDR *S* Typhi away from the null.

In conclusion, Typbar-TCV was effective against culture-confirmed *S* Typhi among children aged 6 months to 10 years in an outbreak setting in Pakistan and was able, with only moderate deployment, to curtail a major XDR *S* Typhi outbreak in a densely populated setting. Our findings show that the vaccine is effective against *S* Typhi irrespective of antimicrobial resistance.

## Data sharing

The crude dataset including all complete individual records cannot be shared because they contain identifiable individual data, including GPS coordinates of the households. Specific additional de-identified data required for systematic review and meta-analysis can be provided upon email request to the corresponding author or first author. The statistical analysis plan, study protocol, and informed consent forms can be made available through email request (to farah.qamar@aku.edu or tahir.yousafzai@aku.edu).

## Declaration of interests

NB-Z received investigator-initiated research grants unrelated to this work from Merck, the Serum Institute of India, and PATH Seattle. NB-Z served on the data safety monitoring board for a study at the University of Oxford, Oxford, UK (*Pancreatic Enzymes and Bile Acids: A Non-Antibiotic Approach to Intestinal Dysbiosis in Acutely Ill Severely Malnourished Children. A Placebo Controlled Double Blind Randomised Controlled Trial in Kenya and Malawi*), and received a nominal fee as a scientific advisory committee member for the COVID-19 Vaccines International Pregnancy Exposure Registry. NB-Z reports two discussions with Bharat Biotech International relating to the design and evaluation of a specific product. This advice was provided pro bono, and under no contractual arrangement other than non-disclosure about the product in question. These discussions are unrelated to the company's typhoid conjugate vaccine that was evaluated in this Article. All other authors declare no competing interests.
