# Supplementary material for: Effectiveness of typhoid conjugate vaccine against culture-confirmed Salmonella enterica serotype Typhi in an extensively drug-resistant outbreak setting of Hyderabad, Pakistan: a cohort study
Source: Lancet Glob Health. 2021 Jul 21;9(8):e1154–62. doi: 10.1016/S2214-109X(21)00255-2 (PMC8315145; doi:10.1016/S2214-109X(21)00255-2)
Supplement: Supplementary appendix [file mmc1.pdf]

THE LANCET
Global Health

Supplementary appendix

This appendix formed part of the original submission and has been peer reviewed. We post it as supplied by the authors.

Supplement to: Yousafzai MT, Karim S, Qureshi S, et al. Effectiveness of typhoid conjugate vaccine against culture-confirmed *Salmonella enterica* serotype Typhi in an extensively drug-resistant outbreak setting of Hyderabad, Pakistan: a cohort study. *Lancet Glob Health* 2021; **9:** e1154–62.

Supplementary Appendix

Typhoid Conjugate Vaccine impact against culture-confirmed Typhoid in Extensively Drug-Resistant Outbreak Setting of Hyderabad, Pakistan: a Case-cohort design

Table 1S: Distribution of study participants

	Surveillance registry N=2,259	Case-cohort N=20,090	Vaccinated cohort N=1,058	Target vaccination N=207,000 (%)
**Taluka**				
Qasimabad	925 (40.9%)	8,068 (40.2%)	719 (68.0%)	68165 (32.9)
Latifabad	1,334 (59.1%)	12,022 (59.8%)	339 (32.0%)	138835 (67.1)
**Gender**				
Male	1,136 (50.3%)	10,530 (52.4%)	548 (51.8%)	101128 (51.5)
Female	1,123 (49.7%)	9,560 (47.6%)	510 (48.2%)	95382 (48.5)
**Age Groups**				
6 M to 59 Months	1,113 (49.3%)	8,555 (42.6%)	500 (47.3%)	115920 (56.4)
60 to 120 months	1,146 (50.7%)	11,535 (57.4%)	558 (52.8%)	91080 (43.6)
**Vaccination By TCV**				
Vaccinated	216 (9.6%)	12,162 (60.5%)	1,058 (100.0%)	207,000 (100.0)
Unvaccinated	2,043 (90.4%)	7,928 (39.5%)	0 (0.0%)	0 (0.0%)

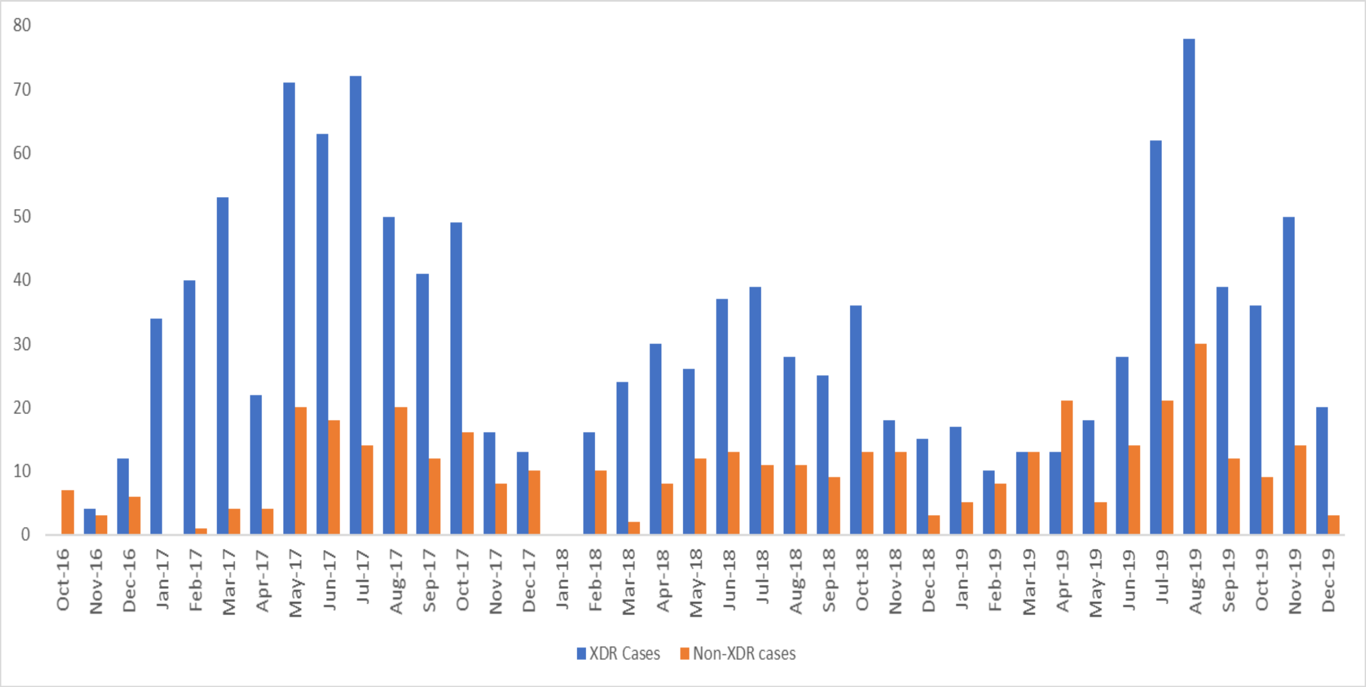

Figure 1S: Epidemic curve XDR and Non-XDR S.Typhi in Latifabad & Qasimabad, Pakistan (October 2016 to December 2019).

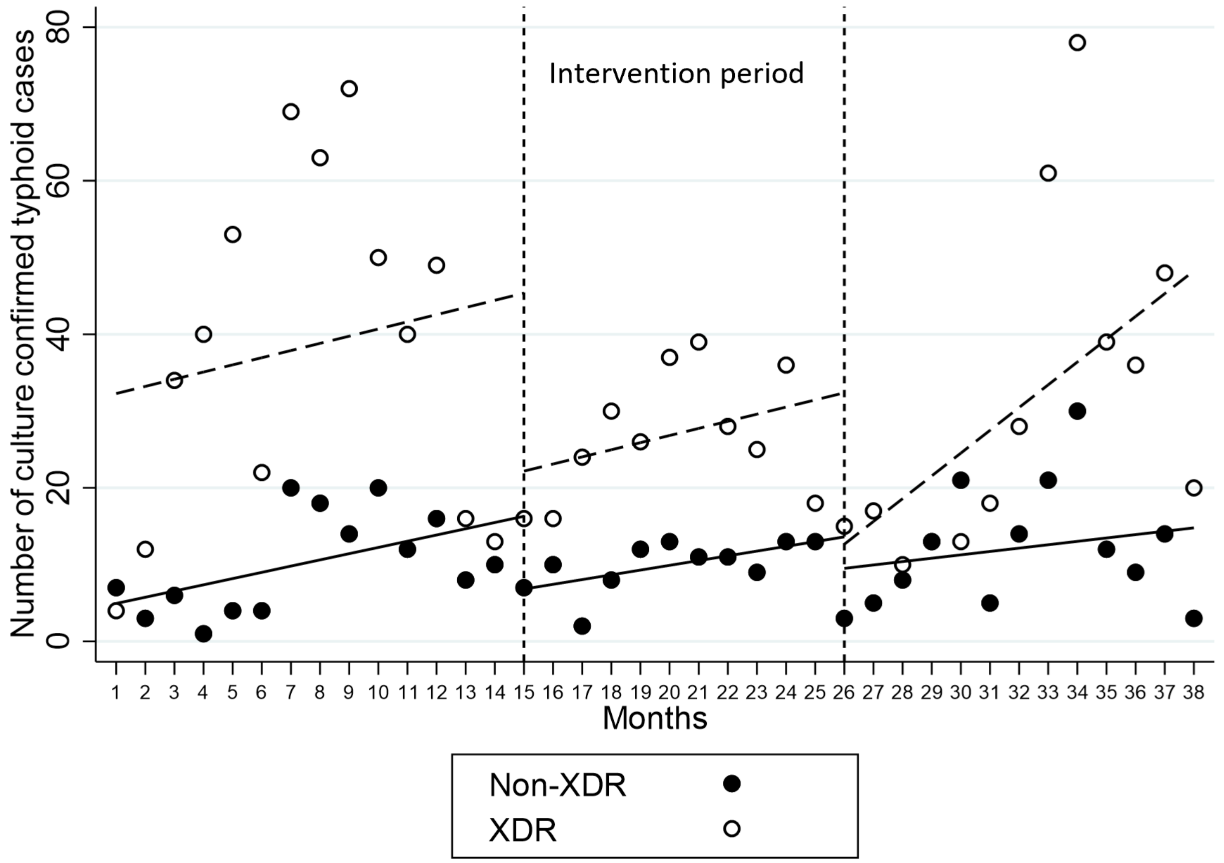

Figure 2S: Number of cultures confirmed XDR and Non-XDR cases from October 2016 to December 2019

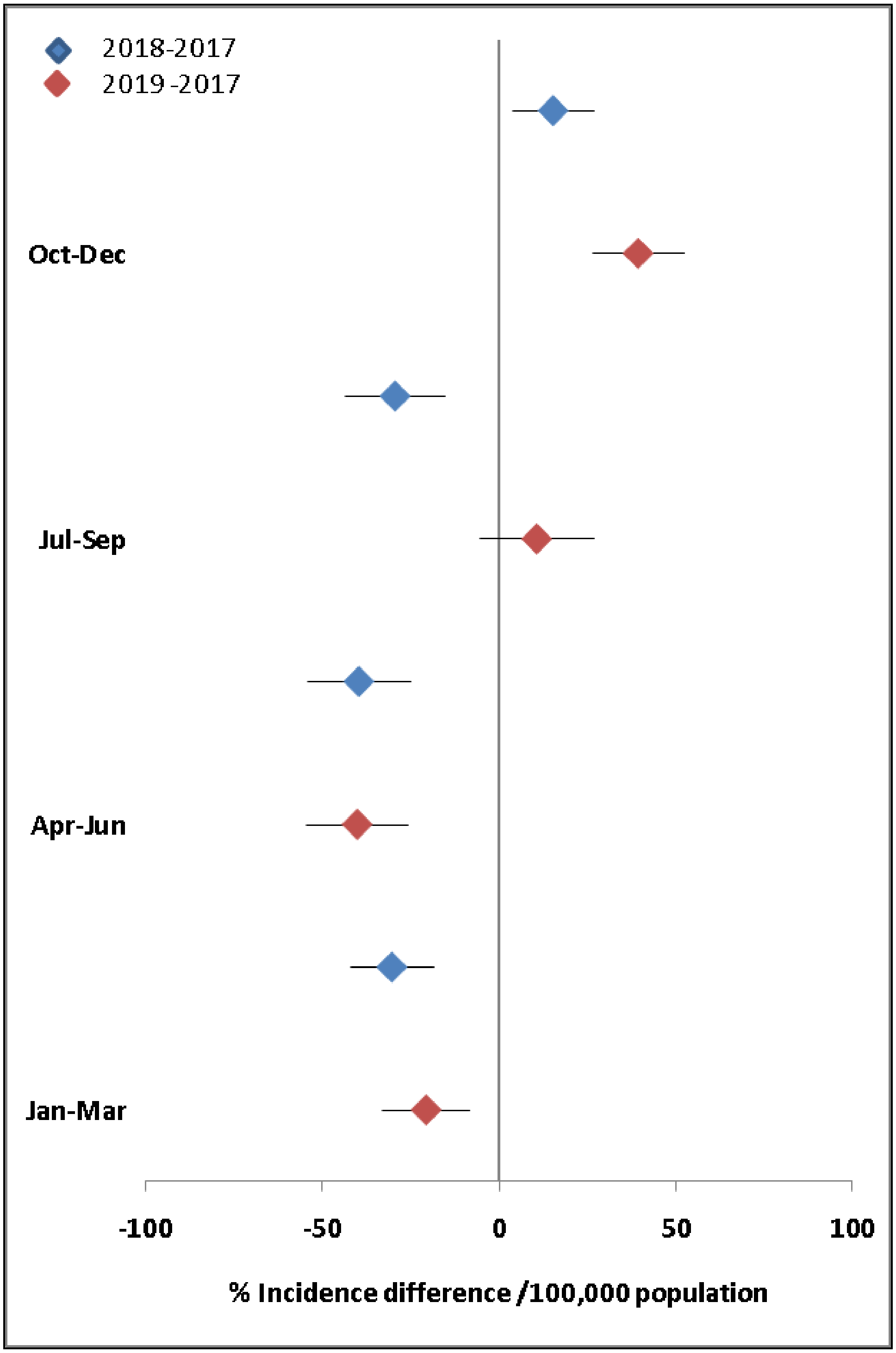

Figure 3S: Impact of Typhoid Conjugate Vaccine (TCV) against culture confirmed typhoid during 2017 – 2019.

Sample size calculation using openEpi

Sample Size:X-Sectional, Cohort, & Randomized Clinical Trials			
Two-sided significance level(1-alpha):			95
Power(1-beta, % chance of detecting):			80
Ratio of sample size, Unexposed/Exposed:			1
Percent of Unexposed with Outcome:			0.46
Percent of Exposed with Outcome:			0.21
Odds Ratio:			0.45
Risk/Prevalence Ratio:			0.45
Risk/Prevalence difference:			-0.25
	**Kelsey**	**Fleiss**	**Fleiss with CC**
Sample Size - Exposed	8282	8281	9064
Sample Size-Nonexposed	8282	8281	9064
Total sample size:	16564	16562	18128

References

Kelsey et al., Methods in Observational Epidemiology 2nd Edition, Table 12-15
Fleiss, Statistical Methods for Rates and Proportions, formulas 3.18 &3.19
CC = continuity correction
Results are rounded up to the nearest integer.
Print from the browser menu or select, copy, and paste to other programs.

Results from OpenEpi, Version 3, open source calculator--SSCohort
